# Current News

**Published:** 1890-10

**Authors:** 


### Current News.

The Missouri State Dental Association.—The twenty-fifth annual meeting of this Association was held at Pertle Springs, Warrensburg, Mo., July 9, 10, 11, 12, 1889, and the following officers were elected for the ensuing year: President, Dr. Henry Fisher, St. Louis; first Vice-President, Dr. J. D. Patterson, Kansas City; Second Vice-President, Dr. J. P. Gray, Sedalia; Recording Secretary, Dr. John G. Harper, St. Louis; Corresponding Secretary, Dr. William Conrad, St. Louis; Treasurer, Dr. James A. Price, Weston, Executive Committee, Dr. J. F. McWilliams, Mexico; Dr. W. L. Reed, Mexico; Dr. W. H. Buckly, Liberty. Board of Censors, Dr. L. M. Nicholson, Fayette; Dr. J. W. Whipple, St. Louis; Dr. J. G. Hollingsworth, Platte City. Committee on Ethics, Dr. N. H. Gaines, Independence; Dr. I. D. Pierce, Kansas City; Dr. C. V. Huff, Knob Nostor. Publication Committee, Dr. H. S. Lowry, Kansas City; Dr. W. E. Tuckey, Butler; Dr. E. W. Stevens, Cameron. Law, Dr. James A. Price, Weston. Supervision of Clinics, Dr. D. J. McMillen, Kansas City.

The next meeting will be held at Pertle Springs, Warrensburg Mo., the first Tuesday following July 4, 1890.

Wm. Conrad,
*Corresponding Secretary.*

321 N. Grand Avenue, St. Louis, Mo.

---

### GRAND UNION MEETING.

There will be a grand union meeting held in Boston, October 28 to 31, inclusive, participated in by the following dental societies under the auspices of the New England Dental Society. The several State societies of the New England States,—The American Academy of Dental Science, Connecticut Valley Dental Society, Harvard Odontological Society, Harvard Dental Alumni Association, Boston Dental College Alumni Association, Boston Society for

Dental Improvement, and the Worcester Dental Society. The committees are arranging for a very enthusiastic meeting. A cordial invitation is extended to all reputable members of the profession to attend.

EDGAR O. KINSMAN, D.D.S.,
*Secretary N. E. D. S.*

15 BRATTLE SQUARE, CAMBRIDGE, MASS.

---

AT the twenty-sixth annual meeting of the Connecticut State Dental Association, held at Hartford, May 20, the following were chosen officers for the ensuing year: President, Civilion Fones, of Bridgeport; Vice-President, E. S. Gaylord, New Haven; Secretary, George L. Parmele, Hartford; Treasurer, Joseph H. Smith, New Haven; Executive Committee, James McManus, Hartford; William H. Rider, Danbury; C. C. Barker, Meriden.

---

THE Twenty-third Annual Meeting of the American Academy of Dental Science will be held in Boston, on Wednesday, November 12, 1890.

The annual address will be delivered by W. W. H. Thackston, M.D., D.D.S., of Farmville, Virginia.

EDWARD N. HARRIS, D.D.S.,
*Corresponding Secretary.*

2 PARK SQUARE, BOSTON, MASS.

---

THERE will be a Union Convention of the Fifth, Sixth, Seventh, and Eighth District Dental Societies in Rochester, N. Y., on the 28th, 29th, and 30th of October.

W. F. ARNOLD,
*Recording Secretary.*

---

DR. E. F. ADAIR, having been consulted about an apparent outgrowth from a left superior canine, dissected away the gum and extracted the tooth, finding attached to it a perfectly formed tooth having a crown with enamel and dentine, and cement on the root; there was also a second similar growth, but much smaller.

